# Decreased *S100A9* expression alleviates *Clostridium perfringens* beta2 toxin-induced inflammatory injury in IPEC-J2 cells

**DOI:** 10.7717/peerj.14722

**Published:** 2023-01-25

**Authors:** Jie Li, Xiaoyu Huang, Kaihui Xie, Juanli Zhang, Jiaojiao Yang, Zunqiang Yan, Shuangbao Gun

**Affiliations:** 1 College of Animal Science and Technology, Gansu Agricultural University, Lanzhou, Gansu, China; 2 College of Life Sciences, Longdong University, Qingyang, Gansu, China; 3 Gansu Research Center for Swine Production Engineering and Technology, Lanzhou, Gansu, China

**Keywords:** *S100A9* gene, Piglet diarrhea, *Clostridium perfringens* type C, *Clostridium perfringens* beta2 toxin, Inflammatory injury

## Abstract

**Background:**

S100 calcium-binding protein A9 (S100A9) is a commonly known pro-inflammatory factor involved in various inflammatory responses. *Clostridium perfringens* (*C. perfringens* ) type C is known to cause diarrhea in piglets. However, the role of *S100A9* in *C. perfringens type* C-induced infectious diarrhea is unclear.

**Methods:**

Here, the *S100A9* gene was overexpressed and knocked down in the IPEC-J2 cells, which were treated with *C. perfringens* beta2 (CPB2) toxin. The role of *S100A9* in CPB2 toxin-induced injury in IPEC-J2 cells was assessed by measuring the levels of inflammatory cytokines, reactive oxygen species (ROS), lactate dehydrogenase (LDH), cell proliferation, and tight junction-related proteins.

**Results:**

The results showed elevated expression of *S100A9* in diarrhea-affected piglet tissues, and the elevation of *S100A9* expression after CPB2 toxin treatment of IPEC-J2 was time-dependent. In CPB2 toxin-induced IPEC-J2 cells, overexpression of *S100A9* had the following effects: the relative expression of inflammatory factors *IL-6*, *IL8*, *TNF-α*, and *IL-1β* was increased; the ROS levels and LDH viability were significantly increased; cell viability and proliferation were inhibited; the G0/G1 phase cell ratio was significantly increased. Furthermore, overexpression of *S100A9* reduced the expression of tight junction proteins in CPB2-induced IPEC-J2 cells. The knockdown of *S100A9* had an inverse effect. In conclusion, our results confirmed that *S100A9* exacerbated inflammatory injury in CPB2 toxin-induced IPEC-J2 cells, inhibited cell viability and cell proliferation, and disrupted the tight junctions between cells. Thus, decreased *S100A9* expression alleviates CPB2 toxin-induced inflammatory injury in IPEC-J2 cells.

## Introduction

Piglet diarrheal disease has seriously affected the economic growth of the pig production industry worldwide. Piglets are highly susceptible to enteritis caused by *Clostridium perfringens* (*C. perfringens*) type C, with a morbidity rate of 100% in infected piglets (Posthaus et al., 2020), and the pathogenicity of this pathogen is mainly mediated by the production of multiple toxins (Forti et al., 2020), among which *C. perfringens* beta (CPB) toxin produced by *C. perfringens* type C is the leading cause of intestinal inflammation (Garcia et al., 2012). *C. perfringens* beta2 (CPB2) is one of the major pathogens of CPB (Schumacher et al., 2013), the original first isolation of CPB2 was from a porcine enteritis strain in 1997 (Gibert et al., 1997). Therefore, the toxin is highly prevalent and can be isolated in piglets with enteritis and diarrhea (van Asten, Nikolaou & Gröne, 2010). It causes fatal enteritis (Schumacher et al., 2013) and plays a crucial role in *C. perfringens* pathogenicity (Nagahama et al., 2015; Uzal et al., 2010). In addition, the CPB2 toxin was highly cytotoxic to HL60, IPEC-J2 and porcine endothelial cells (Gao et al., 2020; Gurtner et al., 2010; Nagahama et al., 2003). Currently, the molecular mechanism of mRNAs in CPB2 toxin-induced damage in IPEC-J2 cells is unclear. Therefore, further genetic analysis is required to identify relevant genes to improve piglets’ ability to resist diarrhea.

S100A9, also known as myeloid-related protein-14 (Mrp14), is a member of the S100 protein family and is an essential pro-inflammatory factor (Huang et al., 2019a; Jhang et al., 2016; Källberg et al., 2012). The protein has been demonstrated to regulate inflammatory responses in multiple cell types and is reported to be up-regulated in various cancer and inflammatory responses (Lee et al., 2017; Markowitz & Carson, 2013; Vogl et al., 2007). In addition, the *S100A9* gene has been reported to perform many biological functions, such as regulating cell proliferation, inducing apoptosis, influencing the migration of inflammatory cells, and activating cell surface receptors (Shabani et al., 2018). S100A9 interactions with other proteins affect the course of virus-induced innate immune responses (Darweesh et al., 2018). *S100A9* can bind to receptors such as TLR4 to activate multiple pro-inflammatory signaling pathways, resulting in an immune response generated by inflammatory factors associated with cell proliferation and inflammatory responses (Kwon et al., 2013; Pandolfi et al., 2016; Shabani et al., 2018). Another study demonstrated the reduced prevalence of inflammatory responses to knocking down the expression of *S100A9* has also been reported (Abers et al., 2021; Zhao et al., 2021). The *S100A9* gene is also involved in the inflammatory immune response caused by a bacterial infection in mammals (Chen et al., 2009). *S100A9* may also play a role in the immunological response to diarrhea caused by *C. perfringens* type C infection, although the exact mechanism is unknown. In a previous study by this research group, the *S100A9* gene was found to play an important role in *C. perfringens* type C diarrheal disease in piglets (Huang et al., 2019b), but the mechanism by which the *S100A9* gene regulates *C. perfringens* resistance in piglets is unknown. IPEC-J2 cells are porcine intestinal columnar epithelial cells that were isolated from newborn piglet jejunum, which are often used as an ideal model for studying pathogenic microorganisms (Brosnahan & Brown, 2012; Liu et al., 2010). In this study, the role of *S100A9* in the immune response has been investigated by overexpressing and interfering with *S100A9* in CPB2 toxin-induced IPEC-J2 cells. This study’s results provide a theoretical basis for further investigations of the molecular regulatory mechanisms of *S100A9* in *C. perfringens* type C diarrheal disease in piglets.

## Materials and Methods

### Ethics statement

The animal experiments were conducted in strict accordance with the regulations of the Administration of Affairs Concerning Experimental Animals (Ministry of Science and Technology, China; revised in June 2004). and had been approved by the Institutional Ethic Committee of Gansu Agricultural University (Approval No. GAU-LC-2018-054). Animals were humanely sacrificed to alleviate suffering.

### Cell and tissue sample collection

The piglets were purchased from Xitai Co., Ltd. in Dingxi, Gansu, China, and raised under the same environmental conditions with natural light and free access to food and water. Thirty seven-day-old piglets (Landrace × Yorkshire) of similar size and weight and in healthy condition were selected. Following the study of Huang et al. (2019b), 25 piglets were randomly selected to receive 1 mL of 1 × 10^9^ CFU/mL *C. perfringens* type C medium orally, and the remaining five piglets were inoculated with 1 mL of sterile medium as a control group, The experiment was conducted for five days. *C. perfringens* type C strain (CVCC 2032) was purchased from the Veterinary Culture Collection Center (Beijing, China). The culture medium was prepared as we described previously (Huang et al., 2019b). Test piglets were divided into control, susceptibility and resistance groups according to the piglet fecal scoring method described previously (Huang et al., 2019c; Yang et al., 2013). After slaughtering under barbiturate anesthesia, the heart, liver, spleen, lung, kidney, duodenum, jejunum and ileum tissues were collected and rapidly frozen and preserved in liquid nitrogen. The porcine IPEC-J2 cell lines were provided by Beina Biotechnology (Beijing, China).

### Cell culture

All cells were cultured in Dulbecco’s modified Eagle’s medium (DMEM; HyClone, Logan, UT, USA) media supplemented with 10% fetal bovine serum (FBS) (HyClone, Logan, UT, USA) and 1% penicillin-streptomycin solution (Gibco, Carlsbad, CA, USA). Cells were incubated at 37 °C in a 5% CO_2_ atmosphere. When the cell confluency reached 80%, 0.25% trypsin solution was used to detach and subculture the cells.

### Transfection and CPB2 toxin treatment

The cell suspension were seeded into plates and transfected when the cells reached a confluency of 70%–80%. Then, referring to the instructions provided in the Lipofectamine® 2000 Reagent (Invitrogen, Carlsbad, CA, USA) manual, chemically synthesized *S100A9* inhibitor negative control (si-NC), *S100A9* inhibitor (si-*S100A9*), mRNA overexpression empty vector (pcDNA3.1), *S100A9* overexpression vector (pc-*S100A9*) were transfected into IPEC-J2 cells and cultured for 24 h, for the knockdown and overexpression of *S100A9*. Finally, the cells were incubated with 20 μg/mL CPB2 toxin for 24 h, and the CPB2 toxin was prepared, purified, and used in doses according to our previous method (Gao et al., 2020; Luo et al., 2020). The *S100A9* overexpression vector was constructed from the pcDNA3.1 cloning vector, with 5′ NheI and 3′ XhoI as cloning sites. It was named pc-*S00A9* and was synthesized by GENEWIZ Life Sciences Company (Suzhou, China). Both *S100A9* inhibitor negative control (si-NC) and *S100A9* inhibitor (si-*S100A9*) were manufactured by Limibio (Hefei, China). The information on interference RNA is shown in Table 1.

**Table 1:** The information of interference RNA sequence.

Vector name	Sense (5′-3′)	Antisense (5′-3′)
si-NC	GGGAUGAGAAAGCCAUAAATT	UUUAUGGCUUUCUCAUCCCTT
si-*S100A9*	UUCUCCGAACGUGUCACGUTT	ACGUGACACGUUCGGAGAATT

### Real-time quantitative PCR (RT-qPCR)

The total RNA was extracted from tissues and cells utilizing TRIzol (TransGen Biotech, Beijing, China) reagent according to the instructions provided in the kit. cDNA was synthesized using the Evo M-MLV reverse transcription master mix kit (Accurate Biotechnology, Hunan, China). RT-qPCR was carried out on a LightCycler 480II instrument (Roche, Basel, Switzerland) with SYBR® Green Premix Pro Taq HS qPCR Kit (Accurate Biotechnology, Hunan, China). The RT-qPCR reaction volume was 20 μL and contained the following: 2×Universal Blue SYBR Green qPCR Master mix-10 μL, Forward and Reverse primers-0.8 μL, cDNA-2 μL, ddH_2_O-6.4 μL. The RT-qPCR reaction conditions were the following: pre-denaturation at 95 °C for 30 s, denaturation at 95 °C for 5 s, annealing at 60 °C for 30 s, 40 cycles. There were three replicates for each group, and the relative expression was calculated using the 2^−ΔΔCt^ method (Livak & Schmittgen, 2001). The relative expression of the mRNA used was calculated with *GAPDH* as the internal reference. The reaction primers were synthesized by Qingke Biotechnology (Beijing, China). The primer information is shown in Table 2.

**Table 2:** A list of the primers used in the RT-qPCR.

Gene name	Transcript no.	Primer sequences (5′-3′)	Length (bp)
*S100A9*	NM_001177906.1	F: GGGACACCCTGAACCAGAAA	193
		R: TCCTCGTGAGAAGCTACCGT	
*IL6*	NM_001252429.1	F: AACCTGAACCTTCCAAAAATGG	90
		R: ACCGGTGGTGATTCTCATCA	
*IL8*	NM_213867.1	F: CTGCAGCTCTCTGTGAGGCTGC	199
		R: TCCTTGGGGTCCAGGCAGACC	
*TNFα*	NM_214022.1	F: GCACTGAGAGCATGATCCG	161
		R: AACCTCGAAGTGCAGTAGG	
*IL-1β*	XM_021085847.1	F: TGATGCCAACGTGCAGTCTA	92
		R: GGAGAGCCTTCAGCATGTGT	
*ZO-1*	XM_021098827.1	F: TGAGTTTGATAGTGGCGTTG	298
		R: TGGGAGGATGCTGTTGTC	
*OCLN*	NM_001163647.2	F: TCCTGGGTGTGATGGTGTTC	144
		R: CGTAGAGTCCAGTCACCGCA	
*CLDN12*	NM_001160079.1	F: ATGACGTCCGTTCTGCTCTT	101
		R: TACGTATGCATGCTGGGAGG	
*GAPDH*	NM_001206359.1	F: AGTATGATTCCACCCACGGC	139
		R: TACGTAGCACCAGCATCACC	

### Cell viability analysis

Cells were seeded into 96-well plates to assess the effect of *S100A9* on CPB2 toxin-induced IPEC-J2 cell viability. The cells were transfected when they reached 70%–80% confluence. After transfection for 24 h, the cells were incubated with 20 μg/mL CPB2 toxin for 24 h. After 24 h, 10 μL cell counting kit-8 (CCK-8) (Beyotime, Shanghai, China) solution was added to every well and incubated for 1 h at 37 °C in a 5% CO_2_, 95% O_2_ incubator. Next, the absorbance of the wells was measured at 450 nm with a microplate reader.

### Inflammatory cytokine protein concentration detection

After transfection and toxin treatment (for 48 h), the supernatant of the treated cells was collected and centrifuged at 2,500 rpm for 20 min, and the supernatant was taken for use in ELISA. Next, the samples were tested using ELISA Kits (Mlbio, Shanghai, China) according to the manufacturer’s instructions. Finally, the OD values were measured at 450 nm using a microplate reader. Then, the standard curve was plotted, and the sample concentrations were calculated and reported in pg/mL.

### Reactive oxygen species (ROS) detection

The amount of ROS in the samples was measured using a ROS assay kit (Solarbio, Beijing, China). The transfected and inoculated cells were collected and suspended in a DCFH-DA (2′,7′-Dichlorodihydrofluorescein diacetate) diluent (10 μmol/L) and incubated at 37 °C, 5% CO_2_ cell incubator for 20 min. To ensure that the cells interact well with DCFH-DA, the cells were mixed by inversion for 3 min, followed by centrifuging at 1,500 rpm for 5 min. The supernatant was discarded, and the excess DCFH-DA was washed with PBS. Next, each experimental group used a fluorescence microplate reader to detect the fluorescence intensity of DCFH-DA. The DCFH-DA fluorescence intensity was directly proportional to the ROS levels in the IPEC-J2 cells.

### Lactate dehydrogenase (LDH) activity assay

The cell supernatant was aspirated after treatment and then centrifuged at 3,500 rpm for 20 min. The activity of LDH in the IPEC-J2 sample was determined by using lactate dehydrogenase detection kits (Jancheng Bioengineering Institute, Nanjing, China).

### EdU (5-ethynyl-2′-deoxyuridine) detection of IPEC-J2 cell proliferation

The number of EdU-positive cells was measured to assess the effect of the *S100A9* gene on CPB2 toxin-induced IPEC-J2 cell proliferation. The collected IPEC-J2 cell suspension was seeded into a 24-well plate and cultured for 24 h. Next, si-NC, si-*S100A9*, pcDNA3.1, and pc-*S100A9* were transfected into IPEC-J2 cells for 24 h using Lipofectamine® 2000. After treatment with 20 μg/mL CPB2 toxin for 24 h, IPEC-J2 cells were incubated with EdU (10 μM) working solution for 2 h (BeyoClick™ EdU-555 Cell Proliferation Assay Kit; Beyotime, Shanghai, China). The nuclei were stained with Hoechst 33342. Qualitative detection was performed using an inverted fluorescent microscope (Olympus, Japan).

### Flow cytometry cycle analysis

After digestion with 0.25% trypsin, IPEC-J2 cells were collected, resuspended in pre-chilled 75% ethanol, and incubated overnight at 4 °C. Next, 2 μL of 10 mg/mL RNAseA was added to the IPEC-J2 cell samples to remove RNA at 37 °C for 30 min. This step was followed by adding 100 μL of 100 μg/mL PI (propidium iodide staining) solution and incubating for 10 min in the dark. Finally, the IPEC-J2 cell samples were detected by a flow cytometer (CytoFLEX, Beckman, CA, USA). Modfit (Verity Software House, Topsham, ME, USA) software was used to analyze the cell cycle distribution data.

### Western blot analysis

The protein from IPEC-J2 cells was collected using radioimmunoprecipitation assay (RIPA) lysate containing 1% phenylmethanesulfonyl fluoride (PMSF). The protein content was determined by utilizing BCA (Bioss, China) protein detection kit. After denaturation, the protein samples were loaded for electrophoresis on an 8% SDS-PAGE gel. The voltages used to run the stacking gel, and the separation gel were 75 and 120 V, respectively. The transferred membrane was blocked by shaking in 5% skim milk (0.5% TBST) at 37 °C for 1 h. The membranes were then incubated with S100A9 antibody (1:1,000; PROGEN, Heidelberg, Germany) or β-actin antibody (1:1,000; Bioss, China) dilution overnight at 4 °C. Next HRP-labeled goat anti-mouse secondary antibody (1:1,000; Servicebio, Wuhan, China) was added and incubated for 30 min. After washing three times with TBST on a decolorizing shaker, the membranes were exposed to a chemiluminescence detection system (Fusion FX; VILBER, Collégien, France). Data was collected, and the quantitative analysis was performed with ImageJ (v1.8.0) software.

### S100A9 protein-protein interaction (PPI) network prediction

The String (Gan et al., 2015) database was used, and evidence was chosen as the meaning of network edges to evaluate the interaction between S100A9 and other proteins. At the same time, *Sus scrofa* was selected as a control organism to predict the medium confidence (0.400) level. The prediction result data were exported to create the protein-protein interaction (PPI) networks between S100A9 and other proteins using Cytoscape (V3.8.0). Betweenness Centrality (BC) was used as a measure. The tightly linked clusters in the PPI network were analyzed to explore the functional clusters using MCODE in Cytoscape software, which clusters play major roles in the PPI networks.

### Statistical analysis

Data analysis was performed using SPSS19.0.The student’s t-test was used for comparison between the two groups. GraphPad Prism 8.0 (GraphPad Inc., La Jolla, CA, USA) software was used for plotting the data. All experiments were repeated thrice. Data are shown as mean ± SD. *P < 0.05 and **P < 0.01 indicate statistically significant differences.

## Results

### Characterization of *S100A9* expression in tissues and cells

**Figure 1:**
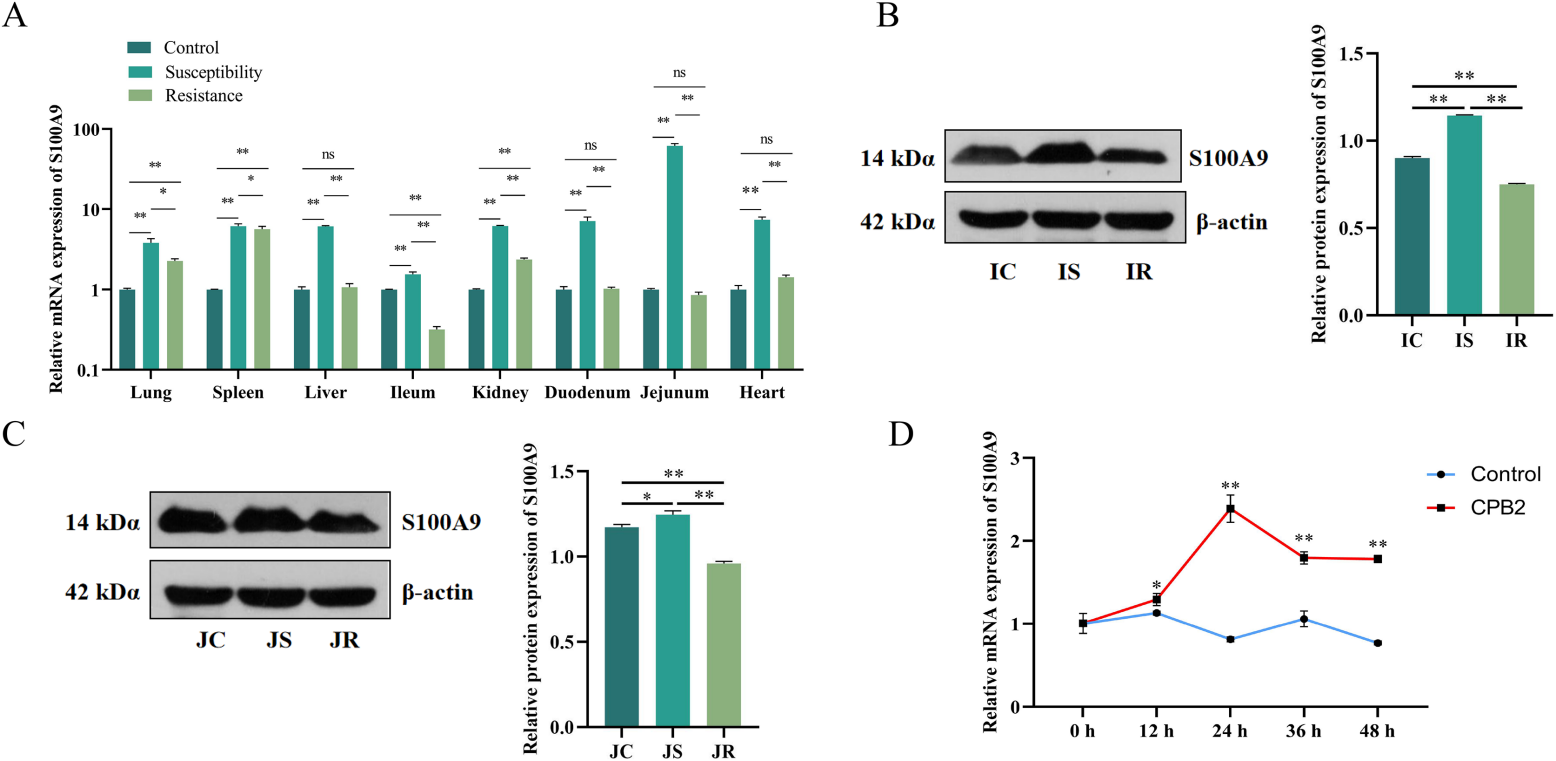
The level of expression of *S100A9* in tissues and IPEC-J2 cells. (A) The relative mRNA expression of *S100A9* in different tissues of each group. The control group represents healthy piglets, susceptibility group represents *C. perfringens* type C susceptible piglets and the resistance group represents *C. perfringens* type C resistant piglets. (B) Protein expression of S100A9 in ileum tissue in the control (IC), susceptibility (IS), and resistance (IR) groups. (C) Protein expression of S100A9 in jejunum tissue in control (JC), susceptibility (JS), and resistance (JR) groups at the protein level. (D) Relative mRNA expression of *S100A9* in IPEC-J2 after different treatment intervals of CPB2 toxin. *P < 0.05, **P < 0.0 1, ns implied *P* > 0.05.

The *S100A9* expression profile and the cellular level were analyzed to investigate the effect of *S100A9* on CPB2 toxin-induced inflammatory injury in IPEC-J2 cells. First, RT-qPCR was used to detect the expression characteristics of *S100A9* in each treatment group of eight tissues. The results indicated that *S100A9* was significantly differentially expressed in the susceptibility group (*P* < 0.05), especially in the intestinal tissues (Fig. 1A). Further, the examination of the protein levels in the ileum and jejunum tissues (*i.e*., the tissues that are closely associated with diarrhea in piglets) by Western blot. The results showed that S100A9 was significantly increased in both IS and JS groups (*P* < 0.01) (Figs. 1B, 1C and S1). The Western blot results indicated that the *S100A9* gene is closely associated with the immune response to *C. perfringens* type C infection. Next, the expression of *S100A9* was examined in different time intervals of CPB2 toxin treatment in IPEC-J2 cells. The results showed that CPB2 toxin significantly induced IPEC-J2 injury starting from 12 h. The expression of *S100A9* was up-regulated in a time-dependent manner and peaked at 24 h of CPB2 treatment after expression stabilized (Fig. 1D). Thus, these results indicated that *S100A9* might play a key in CPB2 toxin-induced IPEC-J2 injury.

### Determining the efficiency of transfection

**Figure 2:**
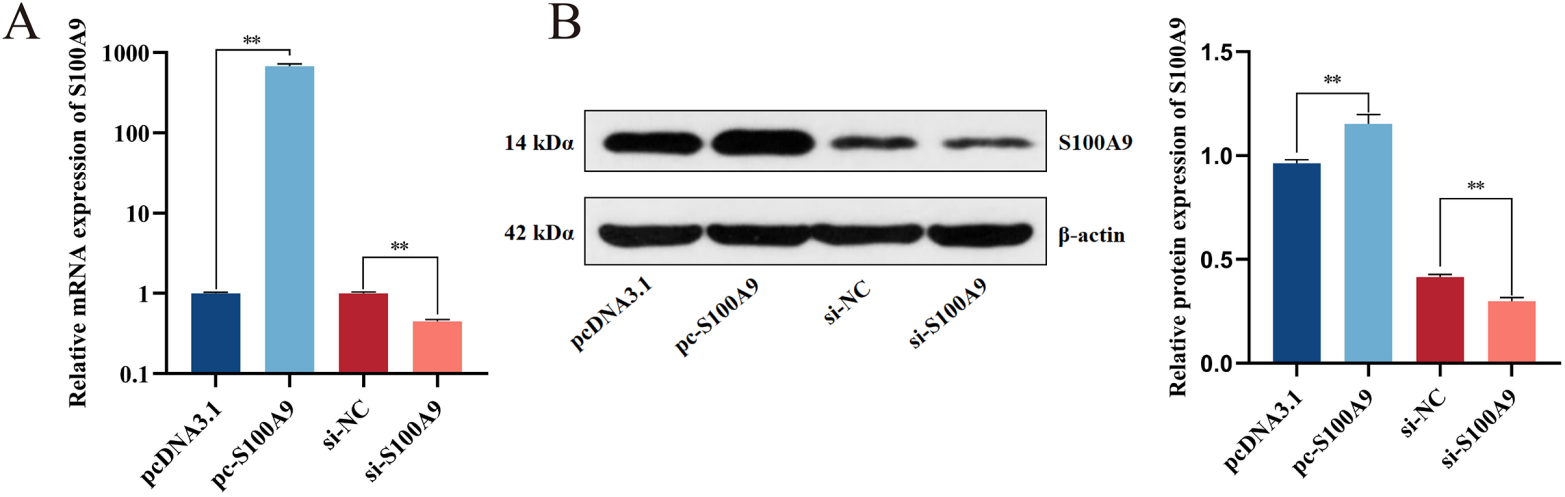
Detection of transfection efficiency of *S100A9*. (A) Relative mRNA expression of *S100A9*. (B) Protein expression of S100A9. *P < 0.05, **P < 0.01.

Chemically synthesized pcDNA3.1/pc-*S100A9* and si-NC/si-*S100A9* were transfected into IPEC-J2 cells. The expression of *S100A9* was detected with RT-qPCR and Western blot after transfection for 24 h. From both mRNA and protein levels, the results confirmed an extremely significant increase in the expression level of S100A9 after transfection with pc-*S100A9* compared with the pcDNA3.1 (*P* < 0.01). Further, expression levels of S100A9 were extremely significantly decreased after transfection with si-*S100A9* compared with the si-NC (*P* < 0.01) (Figs. 2A, 2B and S1), *i.e*., overexpression and interference is success.

### *S100A9* up-regulates the CPB2-induced expression of inflammatory factors in IPEC-J2

**Figure 3:**
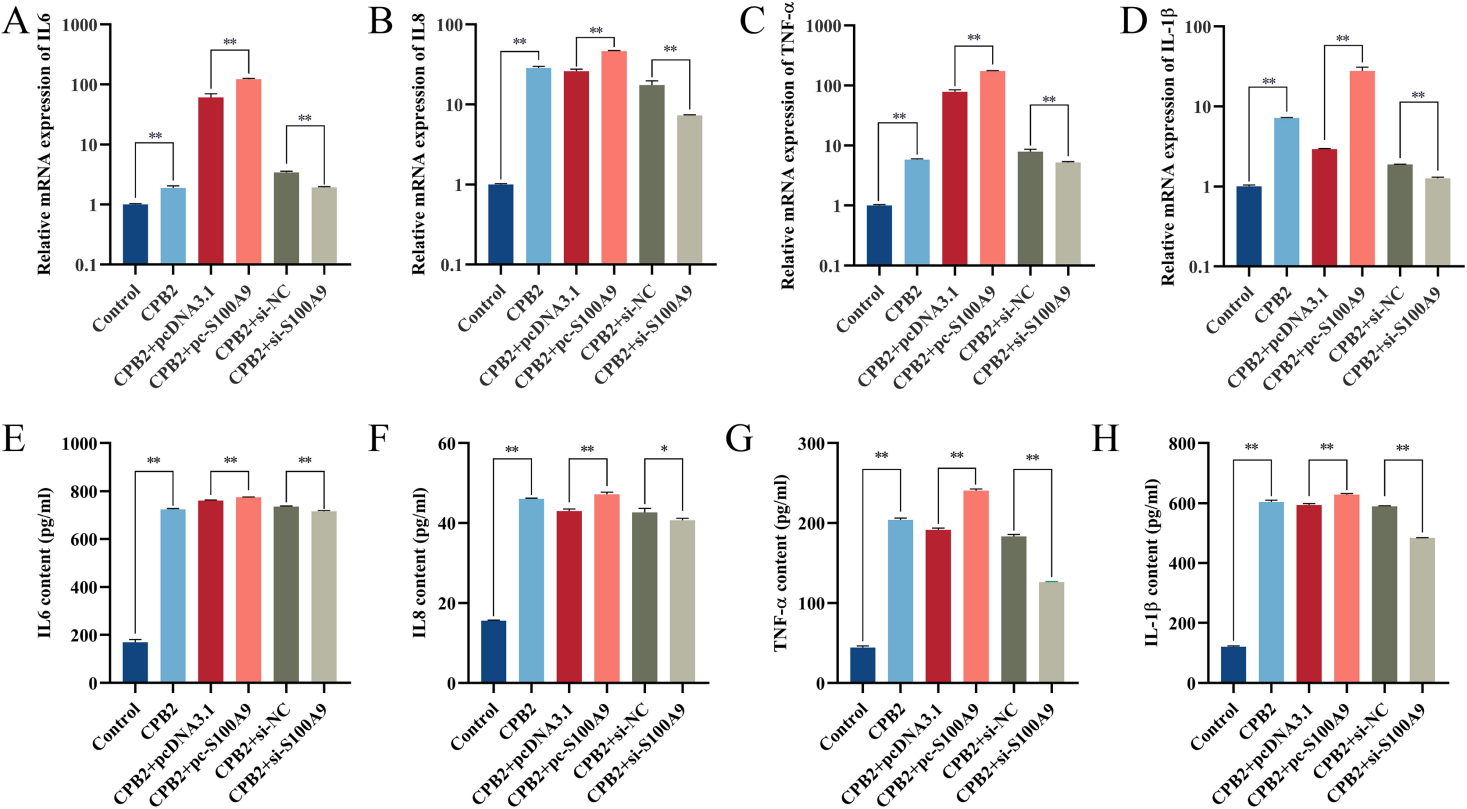
The effect of *S100A9* on the mRNA expression (A-D) and protein content (E-H) of inflammatory factors induced by CPB2 in IPEC-J2 cells. *P < 0.05, **P < 0.01.

RT-qPCR results showed that the expression of *IL-6*, *IL8*, *TNF-α* and *IL-1β* in IPEC-J2 cells induced by CPB2 toxin was significantly increased after overexpression of *S100A9*. (*P* < 0.01). On the other hand, the expressions of *IL-6*, *IL8*, *TNF-α*, and *IL-1β* were extremely significantly decreased in the CPB2 toxin-induced IPEC-J2 cells under interfering with *S100A9* (*P* < 0.01) (Fig. 3A–3D). Further, ELISA was used to detect the levels of inflammatory factors IL-6, IL8, TNF-α, and IL-1β in cell supernatant, the expression level of IL-6, IL8, TNF-α, and IL-1β were significantly increased after transfection with pc-*S100A9* compared with the pcDNA3.1 (*P* < 0.01). However, the reverse trend was observed in the si-*S100A9* group (Fig. 3E–3H). Thus, the results discussed in this section showed that *S100A9* increased the release of inflammatory factors and exacerbated cell injury in CPB2 toxin-induced IPEC-J2 cells.

### ROS and LDH assays

**Figure 4:**
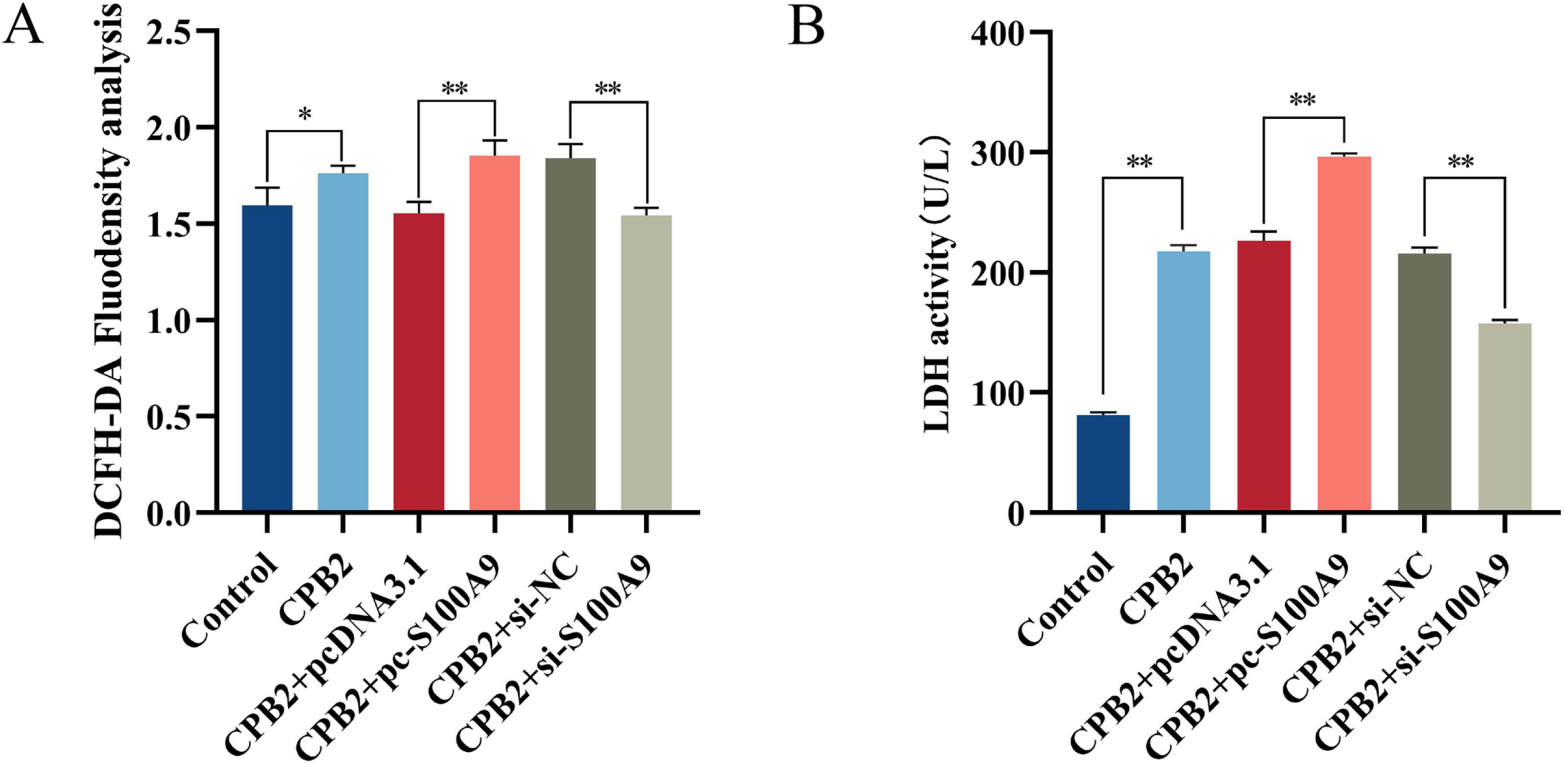
Detection of ROS (A) and LDH (B) in the CPB2 induced IPEC-J2 cells. Stronger fluorescence intensity of the fluorescent probe DCFH-DA implied higher levels of ROS in IPEC-J2 cells. *P < 0.05, **P < 0.01

Cellular ROS levels were measured to investigate further the role of *S100A9* in CPB2 toxin-induced IPEC-J2 cells. The results showed that the fluorescence intensity of the DCFH-DA probe was significantly increased after CPB2 toxin treatment of IPEC-J2 cells compared with the control group (*P* < 0.05). Furthermore, compared with the negative control group, the fluorescence intensity was significantly increased in the *S100A9* overexpression group and significantly decreased in the *S100A9* interference group (*P* < 0.01) (Fig. 4A), indicating that the level of ROS in the cells was elevated after *S100A9* overexpression. Additionally, measurements of the LDH activity cell culture medium revealed that the activity of LDH was significantly increased after CPB2 toxin treatment of IPEC-J2 cells (*P* < 0.01). Furthermore, LDH activity was significantly increased in the *S100A9* overexpression group than in the negative control group. However, the LDH activity was significantly decreased in the *S100A9* interference group (*P* < 0.01) (Fig. 4B). Thus, the results discussed in this section confirmed that *S100A9* promotes cellular damage in CPB2 toxin-induced IPEC-J2 cells.

### *S100A9* inhibits CPB2-induced IPEC-J2 cell viability and cell proliferation

**Figure 5:**
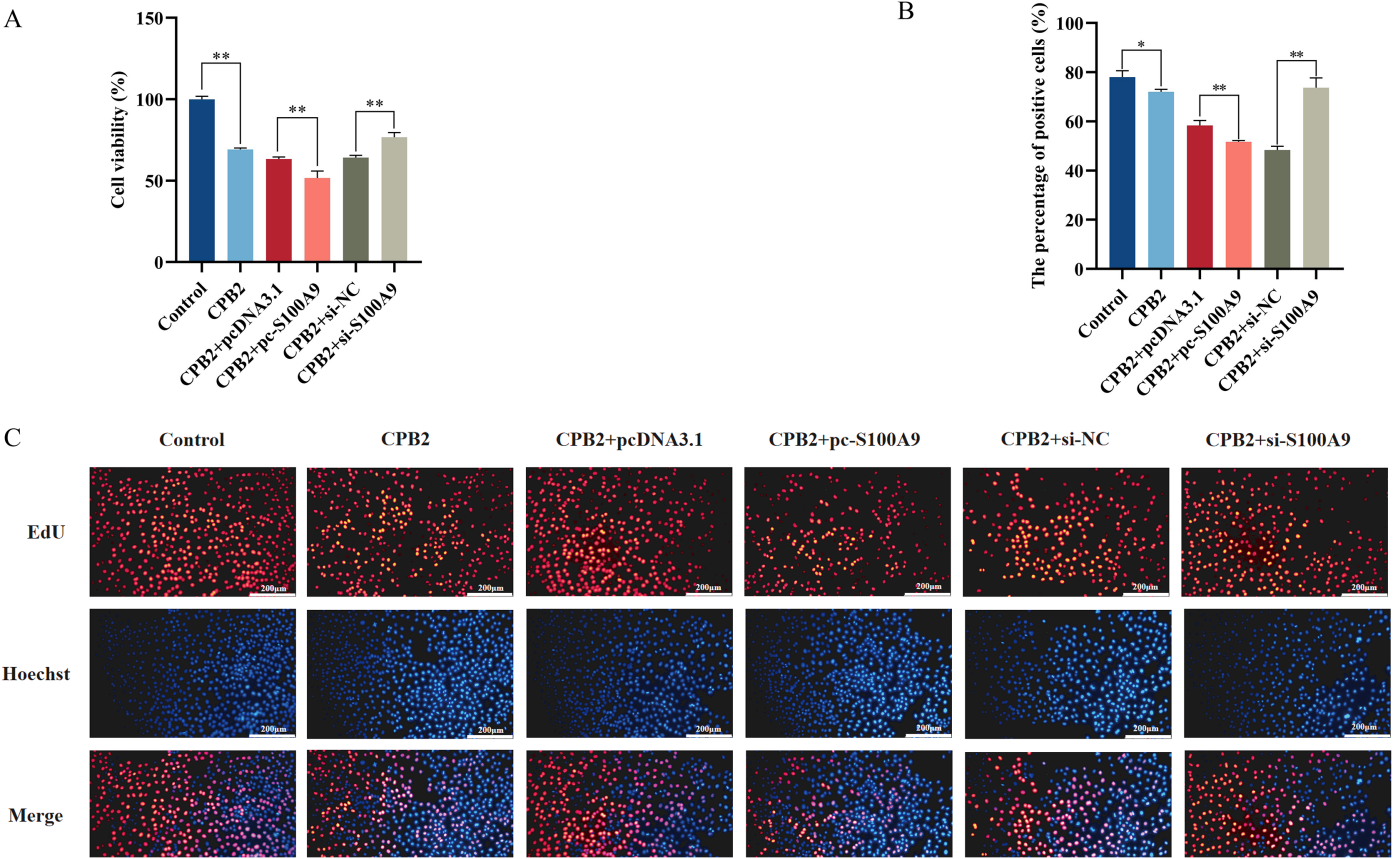
Effect of *S100A9* on CPB2 toxin-induced IPEC-J2 cell viability and proliferation. (A) Cell viability by CCK-8 method. (B) The number of EdU-positive cells. (C) EdU cell proliferation assay. Scale bar: 200 µm. *P < 0.05, **P < 0.01.

The CCK-8 assay was used to measure the cell viability to determine the effect of *S100A9* on CPB2 toxin-induced IPEC-J2 cell viability and cell proliferation. The results demonstrated that the cell viability was significantly decreased in the CPB2 group compared to the control group (*P* < 0.01) and significantly decreased in the *S100A9* overexpression group compared to the negative control group (*P* < 0.01). The reverse trend was observed in the *S100A9* interference group (Fig. 5A). The cell proliferation was detected by the EdU method. The results showed that the number of positive cells in the *S100A9* overexpression group was significantly decreased compared to the negative control group (*P* < 0.01). In contrast, the reverse was observed in the *S100A9* interference group (Fig. 5B). Thus, the results discussed in this section reveal that *S100A9* inhibited cell viability and cell proliferation.

### Effect of *S100A9* on the cell cycle of IPEC-J2 cells induced by CPB2 toxin

**Figure 6:**
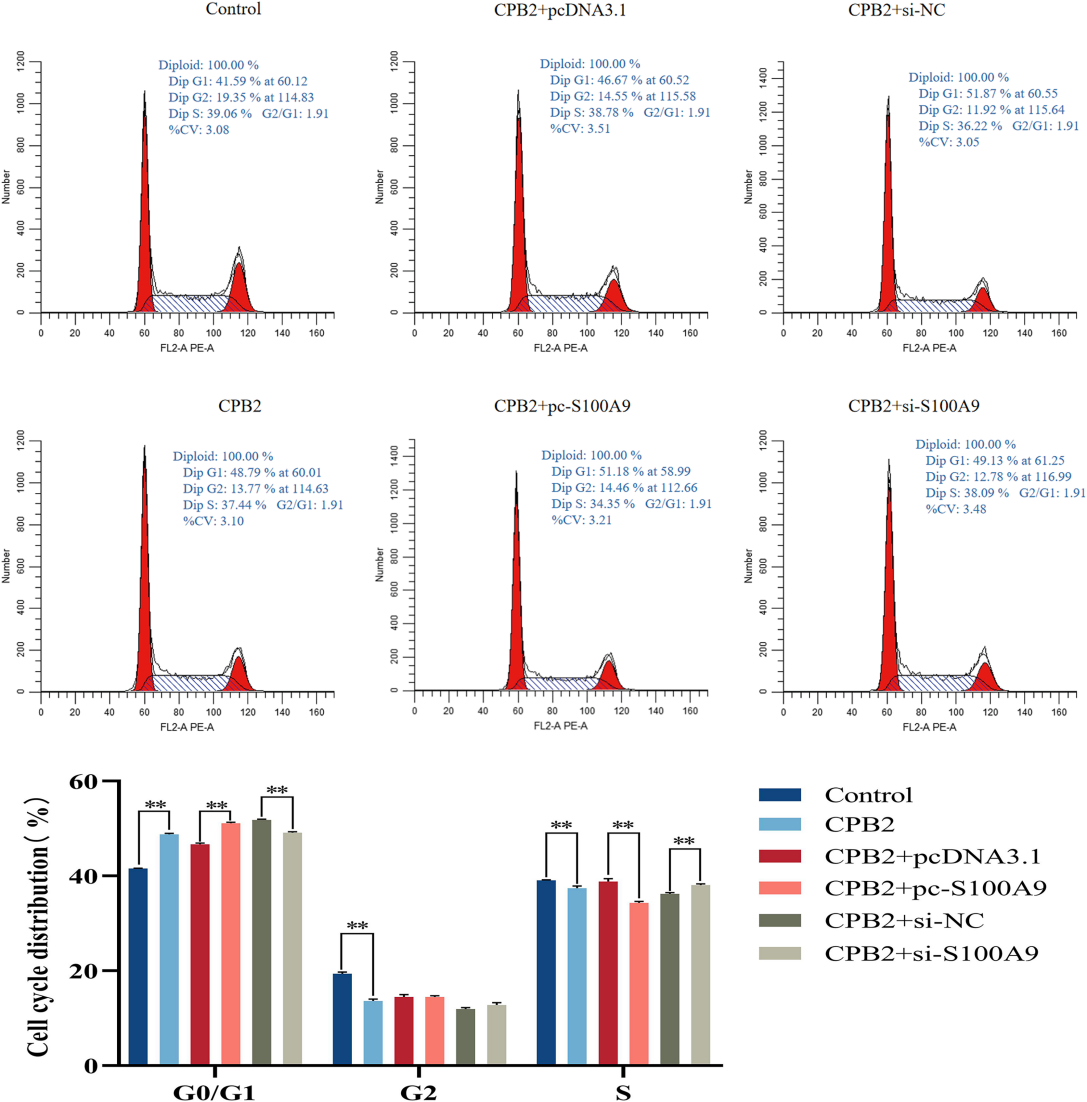
Effects of *S100A9* on the cell cycle of IPEC-J2 cells induced by CPB2 toxin. *P < 0.05, **P < 0.01.

Flow cytometry analysis was used to determine the proliferation mechanism of *S100A9* in IPEC-J2 cells induced by CPB2 toxin. Figure 6 shows the percentage of cells in each experimental group’s G0/G1, G2, and S phases. Compared with the CPB2 group, the percentage of the G0/G1 phase was significantly increased, and the S phase was significantly decreased in the pc-*S100A9* group (*P* < 0.01), while the reverse was observed in the si-*S100A9* group. Thus, the data indicate that the overexpression of *S100A9* prolongs the cell cycle of CPB2 toxin-induced IPEC-J2 cells.

### Effect of *S100A9* on tight junction protein expression in CPB2 toxin-induced IPEC-J2 cells

**Figure 7:**
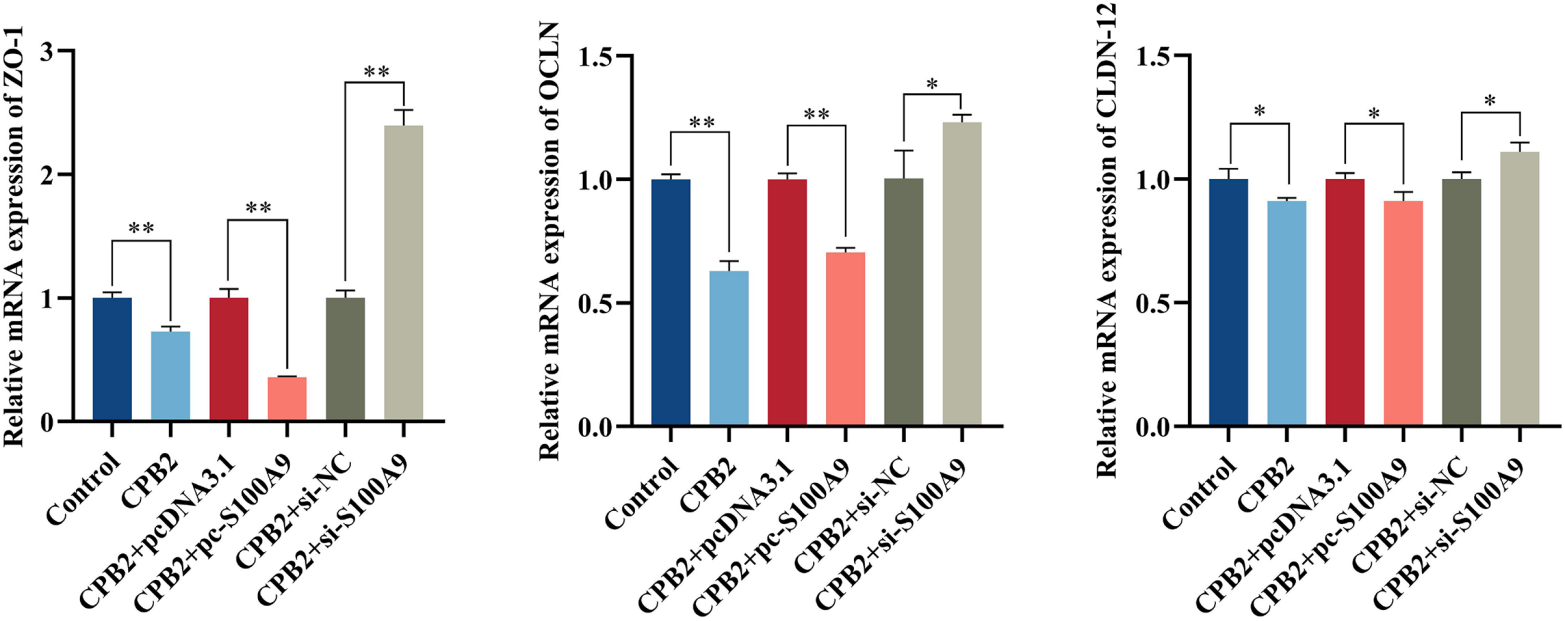
Effects of *S100A9* on CPB2 toxin-induced tight junction protein expression in IPEC-J2 cells. *P < 0.05, **P < 0.01.

The RT-qPCR assay showed that the expression level of tight junction proteins was significantly decreased in IPEC-J2 cells after exposure to CPB2 toxin (*P* < 0.05). In addition, the expression levels of tight junction proteins *ZO-*1, *OCLN*, and *CLDN*-12 were significantly decreased by the overexpression of *S100A9* in the cells. However, inhibition of *S100A9* expression resulted in the reverse trend (*P* < 0.05) (Fig. 7).

### PPI network of immune genes closely related to S100A9

**Figure 8:**
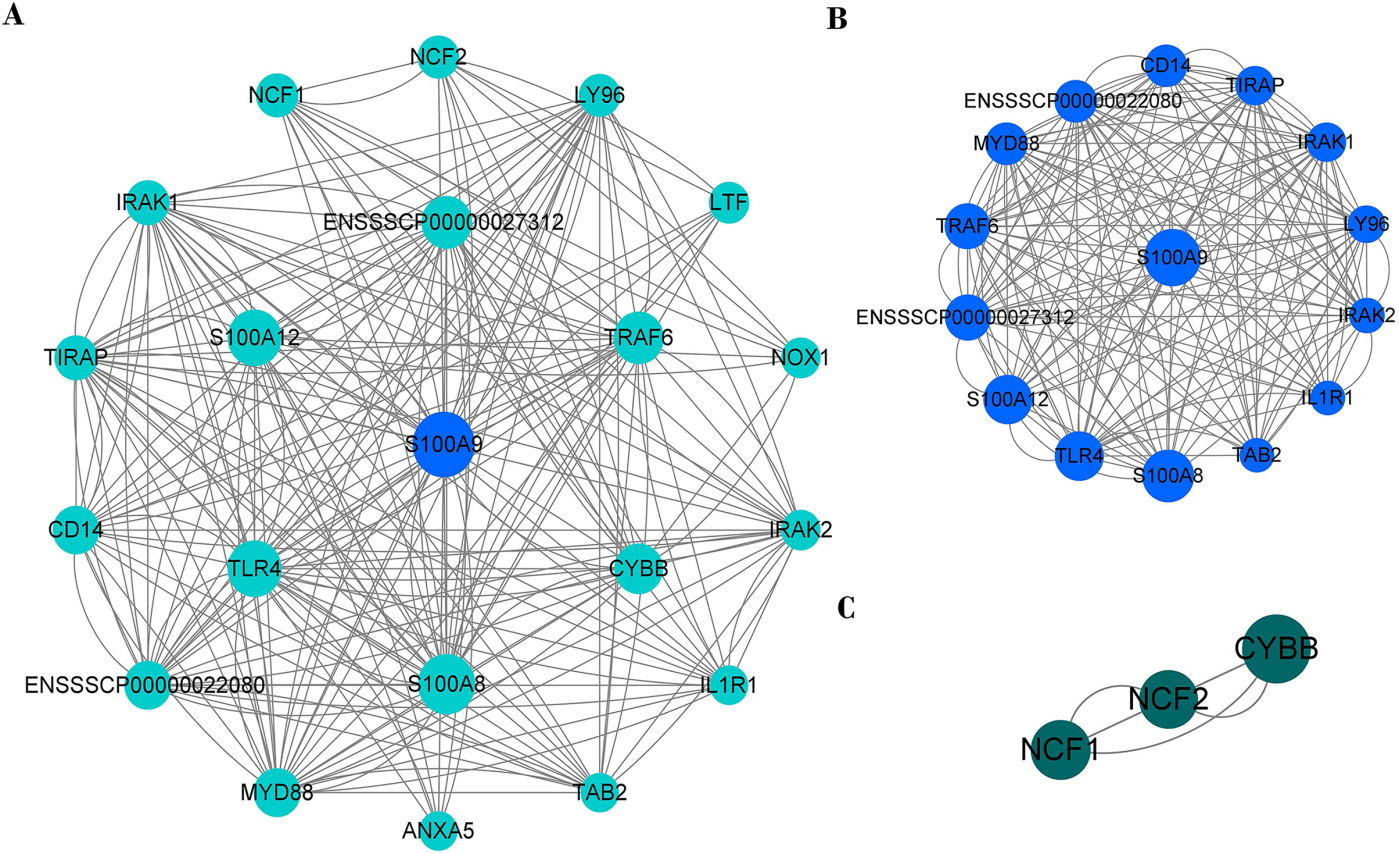
The PPI network of immune genes closely related to S100A9. (A) S100A9 PPI network. (B and C) The main function clusters in the S100A9 PPI network. Larger circles with higher Betweenness Centrality (BC) scores indicate stronger associations with other proteins. A higher number of connecting lines indicate stronger associations.

PPI network analysis shows that there were 20 nodes and 222 edges strongly associated with S100A9 (Fig. 8A). The PPI network was analyzed by the MCODE plugin in Cytoscape (V3.8.0) software, and it was found that there were two clusters of proteins in the PPI network that were closely associated with inflammation, one of which contained 15 nodes and 170 edges (Fig. 8B) and was the most critical functional module. The other contained three nodes and six edges (Fig. 8C). The two protein clusters had scores of 12.143 and 3, respectively. In the PPI network, S100A9, S100A8, TLR4, S100A12, ENSSSCP00000027312, TRAF6 and CYBB proteins had higher BC scores of 31.81, 25.36, 20.98, 20.82, 16.34, 15.89, and 13.72, respectively. Among these, S100A9, S100A8, and S100A12 were pro-inflammatory genes, while TLR4, CYBB, CD14, TRAF6, and other genes played a crucial role in regulating the immune response.

## Discussion

*C. perfringens* type C is a Gram-positive anaerobic bacterium that produces the extremely pathogenic CPB2 toxin (Garcia et al., 2012), causing a variety of inflammatory reactions and infectious diseases. Newborn piglets are susceptible to infection by CPB2 pathogenic bacteria, leading to diarrhea, and are the main cause of piglet mortality. At present, it relies mainly on antibiotics and vaccines, but new methods of molecular resistance to the disease need to be explored. It has been previously reported that *S100A9* affected the innate immune response (Liu et al., 2021). This study revealed that knockdown of *S100A9* reduced injury due to cellular inflammatory injury suggesting that *S100A9* regulates the progression of diarrheal disease. However, the mechanism of action of *S100A9* in CPB2 toxin-induced diarrhea in piglets is unclear.

*S100A9* belongs to the family of damage-associated molecular patterns (DAMPs) (Ehrchen et al., 2009), which promote leukocyte aggregation through a positive feedback regulatory mechanism when the body undergoes an inflammatory response and are often used as a reliable marker for a variety of inflammatory diseases, such as psoriasis, cancer, and rheumatoid arthritis (Christmann et al., 2020; Gebhardt et al., 2006; Huang et al., 2019a). *S100A9* is commonly expressed in various cells, such as colonic epithelial cells, macrophages, and cardiomyocytes (Krenkel et al., 2020; Lee et al., 2012; Li et al., 2019). Additionally, multiple studies have reported that the *S100A9* gene is associated with inflammatory diseases, autoimmune disease and infections (Frohberger et al., 2020; Harman et al., 2020; Huang et al., 2019a). The gene is especially involved in triggering and promoting inflammatory responses, pathogenic infections, and toxin-induced lethality, among other processes (Ehrchen et al., 2009; Frohberger et al., 2020). A study found that increased levels of *S100A9* in inflammatory diseases as a mediator of inflammation and immune response (Simard et al., 2013). Chen et al. (2009) investigated the effect of *Haemophilus parasuis* (HPS) infection on the transcriptome of pig spleen and found that HPS infection resulted in differential expression of genes such as *S100A8*, *S100A9*, and G protein-coupled receptors, with a 21.8 fold up-regulation of *S100A9* gene expression levels. These genes are involved in regulating signaling pathways such as cellular immune response, apoptosis, and cytokine signaling, and the changes in their activity may lead to HPS inhibiting the recruitment of immune cells, lymphocyte activation, and immune response to immune evasion. Another study demonstrated an increased abundance of *S100A9* expression in the intestine of pigs infected with *Campylobacter jejuni* (Negretti et al., 2020). Our study confirmed that the mRNA expression level of *S100A9* was significantly increased in the tissues of piglets susceptible to *C. perfringens* type C. Meanwhile, the expression level of *S100A9* was found to peak at 24 h and then stabilize in IPEC-J2 cells treated with CPB2 toxin, indicating that the damage to IPEC-J2 cells by CPB2 toxin results in an increased expression of *S100A9*. The release of *S100A9* may be caused by increased cellular release of inflammatory factors such as *IL6* and *IL8*, which in turn cause a cascade response in the signaling pathway. si-*S100A9* was found to reduce inflammatory damage in dextran sulfate sodium-treated colonic epithelial cells (CEC), and *IL-6* induced *S100A9* expression in CEC through *STAT3* activation Lee et al. (2012), which confirms our research. Recent studies have demonstrated that the *S100A9* gene has an important role in pig resistance to diarrheal diseases caused by bacterial infections. Vectors were transferred into IPEC-J2 by RNA interference and overexpressing cells produced and subsequently treated with CPB2 toxin. It was observed that when the CPB2 toxin injured IPEC-J2, the levels of *IL-6*, *IL8*, *TNF-α*, and *IL-1β* mRNA and concentration were increased. Further, the release of the inflammatory factors was promoted by *S100A9* overexpression, thereby proving that *S100A9* is a key factor in the process of pig diarrheal disease. Negretti et al. (2020) used a porcine-ligated loop model to study the porcine intestinal response to *Campylobacter jejuni* infection and found that the abundance of inflammatory cytokines *IL*-8 and *TNF*-α were substantially increased during infection. Vincent et al. (2015) used calmodulin in stool as a marker of inflammatory diseases. *S100A9* amplifies inflammatory responses’ and plays a key point in the involvement of organisms in immune-related responses (Vincent et al., 2015). The ROS levels in the intestine can cause various problems (Wang et al., 2021). It has been reported that ROS levels are increased in most inflammatory diseases and that *S100A9* overexpression can result in increased ROS in cells (Shabani et al., 2018). In addition, Zhao et al. (2021) investigated the effect of blocking *S100A9* on LPS-induced lung injury in mice and found that the extent of lung injury was reduced after administration of *S100A9-*specific inhibitors in mice. The results of this study show a significant increase in ROS levels in CPB2 toxin-induced IPEC-J2 cells after overexpression of *S100A9*. Further, there was a decrease in ROS levels after inhibition of *S100A9* was inhibited, indicating that the overexpression of *S100A9* exacerbated the cell damage, consistent with previous reports.

LDH is a stable cytoplasmic enzyme present in all cells, and detection of LDH activity in cell culture supernatant is a common method to evaluate cytotoxicity (Kumar, Nagarajan & Uchil, 2018; Sun et al., 2010) and cell membrane integrity (da Silva et al., 2019; Laev et al., 1993). LDH release indirectly reflects the degree of cellular damage (Kumar, Nagarajan & Uchil, 2018). A previous study found that LDH release was increased in bovine mammary gland epithelial cells induced by *Staphylococcus aureus* (Zhao et al., 2022). Autheman et al. (2013) did a similar study using rCPB to induce primary porcine endothelial cells. In this study, the enzymatic activity of LDH was examined in the cell culture supernatant of each experimental group. The results revealed a significant increase in LDH activity after overexpression of *S100A9*, with a concomitant decrease in LDH activity after inhibition of *S100A9*; this indicates that cytotoxicity increased and exacerbated cell damage after overexpression of *S100A9*. The cell proliferation and viability were assessed by EdU method and CCK8 method, the overexpression of *S100A9* resulted in the inhibition of cell viability and proliferation of CPB2 toxin-induced IPEC-J2 cells; however, inhibition of *S100A9* had a reverse effect. In addition, there was a significant increase in the percentage of G0/G1 phase cells after the overexpression of *S100A9*, which also indicated that *S100A9* inhibited proliferation of CPB2 toxin-induced IPEC-J2 cells. There exists an extensive literature on the effects of *S100A9* on cell proliferation and apoptosis. Zheng et al. (2014) found that *S100A9* has apoptosis-inducing properties on various cells and that recombinant S100A8/9 has stronger apoptosis-inducing activity than *S100A9*. Additionally, calcium-binding proteins is a key enzyme in apoptosis (Zali et al., 2008). The consistent with the results of our study.

Tight junction proteins are known to be closely related to cellular permeability. It has been found that the level of *ZO-1* is significantly reduced when the inflammatory response occurs in the lung, and the PDZ structural domain regulates the LPS-induced inflammatory response in *ZO-1* (Lee, Choi & Song, 2020). Also, tight junction proteins are involved in various intestinal diseases, and their deletion can cause intestinal epithelial dysfunction. Thus, the tight junction proteins play a crucial role in regulating the inflammatory response of the body (Jiang et al., 2021), consistent with our findings. We found that the overexpression of *S100A9* promoted CPB2 toxin-induced injury in IPEC-J2 cells, resulting in a significant decrease in the expression of tight junction proteins. Protein-protein interaction facilitates the functioning, and the prediction of PPI networks is critical for our understanding of biological functions and interactions between cellular components (Athanasios et al., 2017). With the development of various histological techniques, molecular PPI network predictions are becoming more reliable and can predict several diseases by constructing disease models (Tomkins & Manzoni, 2021). The immune response can be predicted based on protein-protein interactions (Mizoguchi & Mizoguchi, 2007). Previous studies have shown that *S100A9* binds to various proteins to regulate immune responses (Björk et al., 2009). The S100A9 has been shown to be a ligand for TLR4 (Björk et al., 2009). TLR4 is expressed in IPEC-J2 cells (Burkey et al., 2009). It was found that *S100A9* could bind to TLR4 to regulate inflammatory response (Raymond et al., 2014). In our study, there was a stronger role between S100A9 and TLR4, which could be another mechanism by which S100A9 exacerbates cell injury, but needs to be verified by further experiments. In addition, TLR4 has been shown to interact with *TIRAP*, *CD14*, and *MyD88* to mediate LPS-induced innate immune responses (Tatematsu et al., 2016). TRAF6 has been reported to mediate various protein-protein interactions that can activate NF-κB and MAPK pathways to regulate immune responses (Walsh, Lee & Choi, 2015). Additionally, due to genetic variants, deletions of *NCF1*, *NCF2*, and *CYBB* can cause hereditary immune diseases (O’Neill et al., 2015). In this study, we constructed a PPI network of S100A9 with other proteins and identified a cluster of proteins, with a score of 12.143, that might play an important role in the inflammatory response.

## Conclusions

In conclusion, this study confirmed that the *S100A9* gene promoted inflammatory injury in CPB2 toxin-induced IPEC-J2 cells, inhibited cellular proliferation, and disrupted cellular tight junctions. Our study revealed the molecular mechanism by which *S100A9* regulates CPB2 toxin-induced IPEC-J2 cell damage.

## Supplemental Information

10.7717/peerj.14722/supp-1 Supplemental Information 1 Full-length images of blots.

10.7717/peerj.14722/supp-2 Supplemental Information 2 Author Checklist.

## Additional Information and Declarations

### Data Availability

The following information was supplied regarding data availability: Raw data are available in the Supplemental Files and Figshare: Li, Jie (2022): Decreased S100A9 expression alleviates *Clostridium perfringens* beta2 toxin-induced inflammatory injury in IPEC-J2 cells. figshare. Dataset. https://doi.org/10.6084/m9.figshare.21225776.v1.
